# Towards transforming malaria vector surveillance using VectorBrain: a novel convolutional neural network for mosquito species, sex, and abdomen status identifications

**DOI:** 10.1038/s41598-024-71856-8

**Published:** 2024-10-10

**Authors:** Deming Li, Shruti Hegde, Aravind Sunil Kumar, Atul Zacharias, Parthvi Mehta, Venkat Mukthineni, Satwik Srimath, Sunny Patel, Maitreya Suin, Rama Chellappa, Soumyadipta Acharya

**Affiliations:** 1 Center for Bioengineering Innovation and Design, Department of Biomedical Engineering, Whiting School of Engineering, The Johns Hopkins University, Baltimore, MD 21218 USA; 2 Department of Electrical and Computer Engineering, Whiting School of Engineering, The Johns Hopkins University, Baltimore, MD 21218 USA; 3 Department of Computer Science, Whiting School of Engineering, The Johns Hopkins University, Baltimore, MD 21218 USA

**Keywords:** CNN, Deep learning, Malaria surveillance, Disease prevention, Epidemiology, Infectious diseases

## Abstract

Malaria is a major public health concern, causing significant morbidity and mortality globally. Monitoring the local population density and diversity of the vectors transmitting malaria is critical to implementing targeted control strategies. However, the current manual identification of mosquitoes is a time-consuming and intensive task, posing challenges in low-resource areas like sub-Saharan Africa; in addition, existing automated identification methods lack scalability, mobile deployability, and field-test validity. To address these bottlenecks, a mosquito image database with fresh wild-caught specimens using basic smartphones is introduced, and we present a novel CNN-based architecture, VectorBrain, designed for identifying the species, sex, and abdomen status of a mosquito concurrently while being efficient and lightweight in computation and size. Overall, our proposed approach achieves 94.44±2% accuracy with a macro-averaged F1 score of 94.10±2% for the species classification, 97.66±1% accuracy with a macro-averaged F1 score of 96.17±1% for the sex classification, and 82.20±3.1% accuracy with a macro-averaged F1 score of 81.17±3% for the abdominal status classification. VectorBrain running on local mobile devices, paired with a low-cost handheld imaging tool, is promising in transforming the mosquito vector surveillance programs by reducing the burden of expertise required and facilitating timely response based on accurate monitoring.

## Introduction

Malaria is arguably one of the largest and most persistent global health challenges in the world, affecting approximately 249 million people annually and resulting in more than 608,000 deaths^1^ worldwide, primarily among children and pregnant women in sub-Saharan Africa. Malaria is a vector-borne infectious disease caused by *Plasmodium* parasites and transmitted by the infected female *Anopheles* mosquitoes^2^. Mosquito vector surveillance—the systematic monitoring of mosquito populations’ species, distribution, behavior, and feeding patterns, together with control interventions, has been a vital strategy to prevent the spread of the disease. Between 2000 and 2015, *Plasmodium falciparum* malaria infection prevalence in endemic Africa halved, and the incidence of clinical diseases fell by 40% through interventions such as long-lasting insecticide nets (LLIN) and indoor residual spraying (IRS)^3^. For effective malaria control, targeted interventions and resource allocation decisions should be driven by a robust vector surveillance system with precise identification of disease vectors. For example, IRS and LLINs are appropriate interventions for *Anopheles funestus*, a primary vector of malaria in Africa, which shows anthropophagic behavior^4^ and mainly bites indoors at night; whereas *Anopheles arabiensis* within the *Anopheles gambiae* species complex is highly zoophagic^4^ and primarily bites outdoors and could account for residual malaria transmission where IRS and LLINs have already been deployed, necessitating alternative strategies. Thus, through vector surveillance, entomologists can pinpoint the high-risk area where malaria transmission is most likely to occur while implementing specific control strategies and preventive measures.

With over 90% of the population at risk^5^, vector surveillance in Uganda, as in most of Sub-Saharan Africa, is a critical component of the national malaria control program. The most prevalent Anopheles are *An. funestus s.l.* and *An. gambiae s.l.*, with a much smaller proportion of other Anopheles also present (as an example in Uganda—other Anopheles species that range from 1 to 10% are *An. coluzzi*, *An. pharoensis*, *An. nili*, etc.). However, given the prevalence, the current entomological surveillance reporting proforma from the districts to the Ministry of Health (MoH) includes the species as the six classes (*An. funestus*, *An. gambiae*, other Anopheles, *Culex*, *Aedes*, and *Mansonia*), sex (female and male), and if female - abdominal status (unfed, fully fed, and gravid). The sex of the mosquito is monitored since only females are capable of biting and potentially transmitting diseases; presence of several males indicates the presence of nearby active breeding sites. Additionally, identifying the abdominal status of mosquitoes can provide insight into their feeding habits as well as human behavior within a given area, helping target interventions more effectively. As an example, an indoor trapping method within a house that yields several mosquitoes with freshly fed abdominal status, indicates the lack of usage of bednets; additionally, mosquitoes (such as *An. funestus*) with gravid abdomens indicates that they survive for a prolonged period after a blood meal to produce eggs- suggesting the ineffectiveness of control measures such as IRS or LLINs, possibly due to emergence of insecticide resistance. These are just a few examples of the type of actionable insights that national malaria control programs attempt to gather on a routine basis, for better, localized, malaria control strategies.

Currently, vector surveillance begins with collecting mosquito specimens at rural sentinel sites using CDC light traps, pyrethroid spray catches (PSC), and sometimes human landing catches (HLC). These specimens are then transported to a central laboratory and morphologically identified based on dichotomous keys^6,7^ by vector control officers (VCOs), individuals trained in entomology and vector surveillance for 3+ years. VCOs analyze the specimens for species, sex, and abdominal status, typically under a light microscope, prior to sending a subset of the collection for molecular identification to confirm morphological identifications. However, the number of individuals with these skill sets is rare and hard to retain in areas with the highest burden of malaria. Despite recognizing the importance of vector surveillance by most National Malaria Control Programs in sub-Saharan Africa, the shortage of expertise and sparse distribution of sentinel sites limits the accuracy of the interventions deployed. There is also a one to 2-month lag between the time of capture of specimens and the time that information is prepared and usable for decision-making, largely due to the expertise bottlenecks, transport time delays, and subsequent manual data upload to national repositories. Therefore, automation using Artificial Intelligence (AI) has the promise of significantly enhancing throughput and accuracy with reduced costs. Additionally, if deployed on simple enough tools, there is a possibility of task sharing this expertise-intensive job with community health workers (CHWs) or volunteers from the rural communities, known as Village Health Teams (VHTs) in Uganda and several other African countries, where surveillance is lacking. Therefore, VCOs can also be empowered to increase the coverage and density of surveillance.

Recently, machine learning, and computer vision approaches have been proposed for detecting and classifying mosquitoes of different species^8^. Visual methods based on feature selection include using features of mosquito body and leg length and color histogram^9^, characteristics of clypeus, proboscis, and wing scales^10^ with support vector machines (SVM), and relevant features from RGB values in the images using random forests (RF)^11^. These methods can obtain above 80% accuracy on specific species of mosquitoes. However, the accuracy is highly dependent on the extracted features, and thus might be limited. In recent years, neural networks (NNs), especially deep learning Convolutional Neural Networks (CNNs), have exceeded in performance compared to previous state-of-the-art machine learning techniques in computer vision^12^. For example, Lorenz et al.^13^ showed that an artificial neural network (ANN), which is composed of many artificial neurons that are interconnected^14^, had better accuracy than traditional discriminant analysis (DA) in the classification of 17 mosquito species based on wing shape characters. More recently, CNN, a type of ANN primarily used to solve visual pattern-recognition tasks^15^, where the network automatically extracts relevant features from images using convolutional kernels, have been used for mosquito species classification. CNNs trained on the mosquito images achieved an 83.9% accuracy at identifying adult mosquitoes from species *Aedes aegypti*, *Aedes albopictus*, and *Culex quinquefasciatus* by Motta et al.^16^. Couret et al.^17^ showed that DenseNet^18^ could distinguish between 16 colonies of mosquito vector species with an accuracy up to 97% and 98% for sex classification. Similarly, You only look once (YOLO)-based algorithm was utilized for high-accuracy species and gender identification of mosquito vectors^19^. To address the open-set problem with novel species detection, Goodwin et al.^20^ designed a multi-tiered ensemble model with CNNs that achieved 97% accuracy for close-set classification of 16 known species and 89% when cascaded with novelty detection. This approach also produced a macro F1 score of 86% for closed-set 39 species classification. By analyzing the behaviors of deep CNNs, Park et al.^21^ found that the models considered the features, such as size, color, and patterns, of certain body parts to discriminate different species of mosquitoes. Alternatively, Kittichai el al.^22^ proposed an image-retrieval system based on deep metric learning to alleviate classification issue such as class imbalance and data scarcity for mosquito species identification. Finally, Zhao et al.^23^ trained a Swin transformer^24^ model has a performance of 99% accuracy for 17 mosquito species identification on a high-definition data set taken with the digital single-lens reflex (DSLR) camera and macro lens.

The aforementioned works^13,16,17,20,21,23^ have demonstrated the utility of deep learning CNNs for various aspects of mosquito species identification. However, several constraints prevent them from directly impacting ongoing malaria surveillance programs, especially in Africa. Effectiveness and generalizability on fresh wild-caught specimens are the first limitations. Most of these models were trained on images of colony-bred or desiccated mosquitoes and focused only on species classification. Uganda, like most African countries, has a system of vector surveillance that is very specific to identifying and counting the commonly prevalent species (six categories as mentioned previously), sex, and abdominal status, and reporting it centrally based on a standardized reporting proforma mentioned earlier; For an AI-based tool to be applicable in field conditions, algorithms need to be developed to mimic all three parameters. Second, most of the available literature analyzed datasets typically imaged using microscopes, thus limiting their accessibility and scalability to district and community-based programs. The image-retrieval system^22^ that is likely to be accessed through cloud during inference time requires the internet connection. To be translated into actionable products be integrated into local malaria surveillance, the solution should ideally to be AI algorithms/apps that run on low-cost devices like basic smartphones and need to operate in internet-denied or spotty internet connectivity. Third, another limitation of these approaches, whose parameter count ranges from 20.2M^18^ to 197M^24^, is deployability. To perform three separate tasks, it would require three individual models. The lower-cost smartphones commonly used by community health programs in Africa, which generally do not have the memory or computational power comparable to expensive edge computing devices. Therefore, the model should be as small and fast as possible, ideally in a multi-task fashion, to be deployed on mobile devices. 

To address the above concerns, in this paper, we propose a novel pipeline to mosquito identification by integrating lightweight models that can run locally on inexpensive smartphones, allowing for the direct capture of images of mosquitoes and almost instantaneous result feedback in the field. Instead of focusing on one task at a time, our multi-task approach features classifications of not only mosquito species but also the sex and abdomen status of a mosquito concurrently, which replicates the complete data that need to be reported to the MoH as part of the existing vector surveillance workflow. Our model shows promising results of classifications in an efficient way, demonstrating its feasibility to streamline mosquito classification and surveillance in resource-constrained settings without access to an internet connection. A pilot study at various locations in Uganda is underway by deploying our proposed method with an live app in the hands of VHTs to prove the effectiveness of our approach compared to conventional surveillance, potentially task-sharing malaria vector surveillance in Uganda and potentially in other countries like Zambia and Ghana in East Africa.

**Fig. 1:**
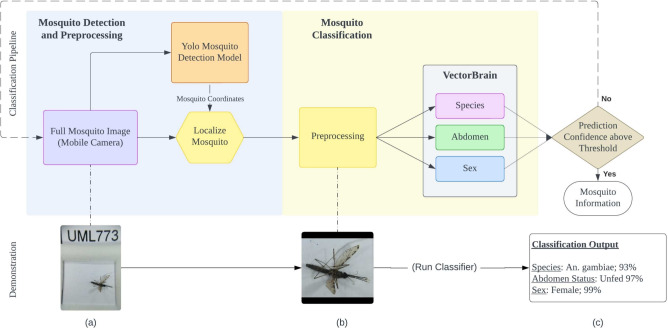
This image provides a flow chart of the classification pipeline along with an example that showcases the various stages when classifying an image of a mosquito. The first stage, (**a**), shows the full image of the mosquito that needs to be classified. In the second stage, (**b**), a YOLO algorithm has been applied to crop the image of the mosquito using its coordinates, and a series of image transformations have been applied to prepare the image for classification. These transformations include resizing the image to a specific dimension and normalizing the pixel values using the ImageNet dataset mean and standard deviation. Finally, in stage (**c**), the output of the classification algorithm is visible, which determines the type of mosquito present in the image. These three stages demonstrate the full image processing that takes place in order to classify a specimen accurately.

## Results

### System overview

Illustrated in Fig. 1, All images of mosquitoes were captured on a tray using a mobile phone and a 15X clip-on macro lens. The significant whitespace, tray, and specimen ID in the photograph necessitates the first stage of our algorithm- detecting and cropping the mosquito body using a real-time detection model—You Only Look Once (YOLO) model^25^. In the second step, the images of localized mosquitoes are processed by VectorBrain, a multi-task EfficientNet^26^, specifically tailored for mosquito classification with concurrent outputs for species, sex, and abdominal status. The results are described on a large dataset of mosquito images taken from freshly collected specimens from vector surveillance field sites in Uganda, Zambia, and Ghana using PSC, CDC light traps, and HLC. Our results show that the proposed pipeline can achieve high accuracy and efficiency in mosquito classification tasks typical to vector surveillance programs in Africa (Species, Sex, Abdominal status), making it a promising tool for mosquito surveillance and control efforts.

### Mosquito detection and classification accuracy

**Fig. 2:**
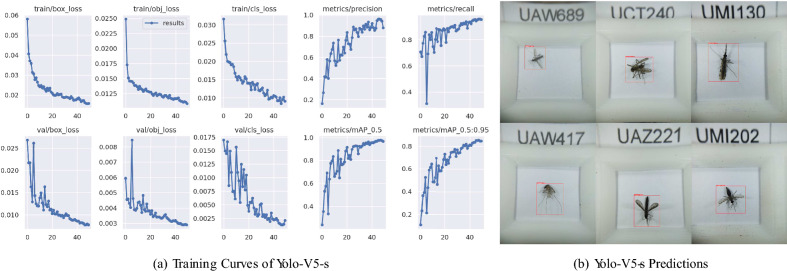
The training process and qualitative results of step one of the algorithm- that of detecting the mosquito within the image- using YOLO V5 small. In (**a**), the x-axis denotes the number of epochs during training. The box loss measures how ”tight” the predicted bounding boxes are to the ground truth object. The object loss measures whether the ground truth object is detected i.e., exists at an anchor. The class loss measures the correctness of the classification of each predicted bounding box. Precision, Recall, mAP at 0.5 IoU, and mAP for IoU from 0.5 to 0.95 curves are also included in the figure. (**b**) shows few examples of qualitative results of predicted bounding boxes on testing specimens.

**Fig. 3:**
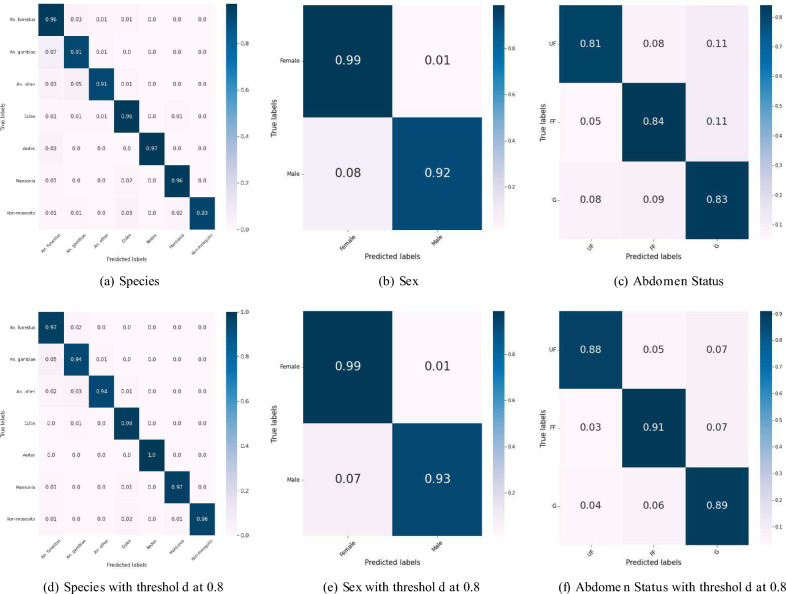
Confusion matrices with accuracy for species, sex, and abdominal status classifications. The plots (**a**), (**b**), and (**c**) in the first row report the accuracy on the 5-fold cross validation results by aggregating all test folds. In (**a**), the supports are: *An. funestus*—3264, *An. gambiae*—1730, *An. other*—906, *Culex*—2028, *Aedes*—32, *Mansonia*—671, and *Non-mosquitoes*—183. In (**b**), the supports are: female—4619, and male—1101. In (**c**), the supports are: UF—1576, FF—790, G—776. The plots (**d**), (**e**), and (**f**) are the confusion matrices on the same sets of data with a threshold of 0.8 omitting around 5% of the data.

As in the first stage of our proposed pipeline, we employed YOLOv5 small, which is lightweight and fast, to localize the mosquito in the images and used the detected coordinates to crop the part of the image only containing the mosquito body. By doing so, the effective resolution of the mosquito, as seen by the next stage of the algorithm, is maximized, and the background is minimized. To illustrate the detection performance of the YOLO, Fig. 2a reports precision, recall, and mean Average Precision (mAP) logs during training and validation. The model with the best validation performance achieved a precision of 96.00%, recall of 90.50%, mAP at 0.5 Intersection over Union (IoU) of 95.87%, and mAP for IoU from 0.5 to 0.95 of 85.69%. Figure 2b contains the visualized results of the detected bounding boxes and confidence values on a batch of test data. The bounding boxes were annotated to include all body parts of the mosquitoes while maintaining tightness. Consequently, the YOLO model is trained to ensure that no part of the mosquito such as legs and wings, which are crucial for classification tasks, is dismissed and maximize the ROI in the cropped images.

Classifications of species, abdomen, and sex on the cropped images were done by VectorBrain with a common feature extractor and separate output branch for each task, detailed in the Method Section. Confusion matrices of mosquito classification in species, sex, and abdomen status are shown in Fig. 3. A 5-fold stratified cross-validation was performed on 4195 fresh field-caught specimens mostly imaged on the day of trapping (species-specific breakdown in Table 5). Although in some instances multiple images might have been taken of the same mosquito (by rotating, or flipping the specimen between photographs), a strict separation was maintained between the actual specimens used for training, validation, and testing. As shown in Fig. 3a,b,c, the species classification model achieved an overall accuracy of $94.44\pm 2\%$. Similarly, the sex classification model achieved an accuracy of $97.66\pm 1\%$, and the abdominal status classification model achieved an accuracy of $82.20\pm 3.1\%$. These results are original accuracies, without any thresholding, a strategy we devise to remove low-confidence candidates from model outputs, which we will explain in the Evaluation Subsection.

**Fig. 4:**
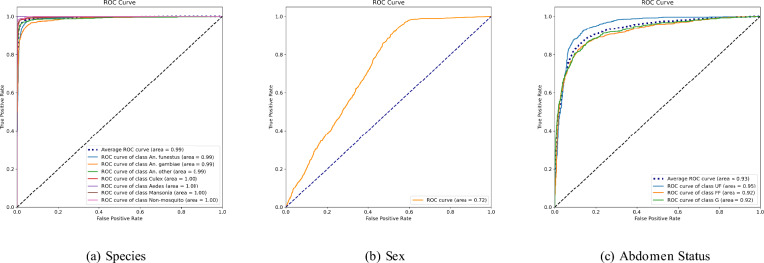
ROC curves for species, sex, and abdominal status classifications.

Table 1 summarizes the performance of the classifications in F1 score based on precision and recall for each class, the macro-averaged total F1 score, and the weighted averaged F1 score. The macro-averaged F1 score assigns equal importance to every class, regardless of the number of data points in each class. This method is particularly useful when dealing with imbalanced datasets. On the other hand, the weighted-averaged F1 score also considers the number of samples in each class, thus being useful when the performance of both minority and majority classes is important. For species classification, the F1 scores for each class are as follows: *An. funestus* ($96.00\% \pm 2\%$), *An. gambiae* ($91.23\% \pm 2\%$), *An. other* ($91.70\% \pm 3\%$), *Culex* ($96.00\% \pm 1\%$), *Aedes* ($97.05\% \pm 2\%$), *Mansonia* ($96.21 \% \pm 1\%$), and *Non-mosquito* ($93.56 \% \pm 3\%$). The overall macro-averaged F1 score is $94.10\% \pm 2\%$, and the weighted-averaged F1 score is $94.40\% \pm 1.7\%$. Regarding sex classification, the F1 scores for the female and male classes are $99.01\% \pm 1\%$ and $93.03\% \pm 2\%$, respectively. The overall macro-averaged F1 score is $96.17\% \pm 1\%$, and the weighted-averaged F1 score is $97.67\% \pm 1.5\%$. For abdominal status classification, the F1 scores for the unfed, fully fed, and gravid classes are $86.00\% \pm 3\%$, $80.42\% \pm 4\%$, and $77.89\% \pm 3\%$, respectively. The overall macro-averaged F1 score is $81.17\% \pm 3.1\%$, and the weighted-averaged F1 score is $81.98\% \pm 2.8\%$. Class-wise and average receiver operating characteristic (ROC) curves^27^ for species (AUC=0.99), sex (AUC=0.72), and abdomen (AUC=0.93) classifications are shown in Fig. 4. These results prove the high accuracy and effectiveness of the developed models in mosquito classification using mobile phone images, which hold promise for enhanced mosquito surveillance and control efforts. We also evaluated the impact of setting a confidence score threshold whereby specimens with a lower prediction confidence could be further evaluated for image blurriness, disadvantageous anatomical position- such as wings not being visible etc. Such an approach could be implemented in a field tool/ app, that can either offer an identification if the confidence score is above a certain threshold, or require the user (such as a field entomologist) to change the orientation of the specimen and retake the picture. Figure 3d,e,f show that there is a 3% increase in accuracy from 94 to 97% if we set a confidence threshold of 80%. This results in around 5% of specimens being ‘discarded’—which in a closed loop app, would be a trigger to retake the image immediately. Figure 7 shows the tradeoff between accuracy and the number of specimens ‘discarded’ as we increase the confidence score threshold. For this algorithm to be translated into a field deployed app, an optimal threshold is chosen to improve accuracy without severely burdening the field entomologists with the number of ‘retake please’ prompts.

**Table 1:** Accuracy (micro-averaged F1 score), macro-averaged F1 score, and weighted-averaged F1 score of mosquito classification in species, sex, and abdomen status using 5-fold cross validation.

Results	Accuracy (avg (min–max))	Macro-averaged F1 score (avg (min–max))	Weighted-averaged F1 score (avg (min–max))
Species	94.44 (93.86–96.04)%	94.10 (92.43–95.20)%	94.40 (92.12–96.00)%
Sex	97.66 (96.21–98.09)%	96.17 (94.77–96.950)%	97.67 (96.20–98.30)%
Abdomen	82.20 (79.80–84.55)%	81.17 (77.03–83.56)%	81.98 (79.54–83.98)%

### Comparative analysis of alternative frameworks

**Table 2:** Comparison of YOLOv5 and Faster R-CNN models in the number of parameters, model size, mAP at 0.5 IoU, and time to 100 images on an AMD Ryzen 7 3700x CPU.

Model	Number of parameters	Trained model size	mAP at 0.5 IoU	Time to run (100 images)
YOLOv5 Small	7.7M	27.4 MB	95.9%	2.8 s
YOLOv5 Large	58.8M	216.1 MB	98.0%	8.7 s
YOLOv5 Nano	1.3M	4.7 MB	89.2%	1.5 s
Faster R-CNN	42.5M	162.1 MB	95.8%	13.3 s

For mosquito detection, we employed the YOLOv5, based on size-performance optimization^25^ and due to its several advantages over other object detection models, such as Faster R-CNN. One advantage of YOLOv5 is its ability to perform object detection with a single network evaluation, making it much faster than R-CNN and Fast R-CNN models. Additionally, YOLOv5 examines the entire image during testing, which enables its predictions to be informed by the global context in the image rather than just the local features of a region of interest, as in other models. According to Table 2, YOLOv5 Small is the most suitable model for our application considering the trade-offs between size, running time, and accuracy. YOLOv5 Small has 94.9% mAP and 7.7M #Params. It is 27.4 MB in model size such that it only takes 2.8 s to run 100 mosquito images on our CPU. Even though YOLOv5 Nano is smaller in size and runs faster, its accuracy is more than 5% less than that of YOLOv5 Small. Notably, YOLOv5 Large and RCNN models have higher accuracy but require more computational resources and longer running time, which makes them less feasible for running on low-end devices.

**Table 3:** Comparison of efficientNet-B1 with other classification models.

Model	Number of parameters	Model size (MB)	Accuracy (%)
EfficientNet-B1	7.8M	26.6 MB	94.03
EfficientNet-B4	19M	76.1 MB	94.80
ResNet-18	11.2M	42.9 MB	92.02
MobileNetV2	3.4M	13.1 MB	92.72
DenseNet-121	8.1M	31.2 MB	93.80
Vision transformer (ViT) large	301M	1.2 GB	87.90
YOLO v5 (small)	7.7M	27.4MB	85.50

When building our mosquito classification algorithms, extensive comparisons of different classification models are made based on the number of parameters, model size, and accuracy. The requirement for our classification model is to be able to run on basic smartphones with minimum size and high accuracy. According to Table 3, compared to ResNet-18^28^ and DenseNet-121^18^, EfficientNet-B1 achieves both better accuracy and smaller size. Even though MobileNetV2^29^ is smaller in size and has fewer parameters, EfficientNet-B1 excels in classification accuracy. Vision Transformer (ViT) large^30^ does not accurately perform on par with EfficientNet-B1. It could be due to the following reasons 1. The features in differentiating mosquito species are very fine-grained. However, ViT splits the input images into visual tokens, which are $16\times 16$ patched in this case, whereas CNNs directly extract features from individual pixels. 2. The number of data samples might not be enough to train a ViT sufficiently. In addition, EfficientNet-B4 achieves the highest accuracy in our experiments but at the expense of about three times more parameters and a larger size. We also explored the possibility of deploying YOLO as a one-step detector classifier. We performed the same analysis on the mosquito classification as other classifiers, which produced a classification accuracy 8.5% lower than EfficientNet-B1. It is beneficial to use a single YOLO for both tasks, resulting in a simpler workflow and less memory consumption, but in order to achieve the best accuracy for vector surveillance, we chose EfficientNet-B1 as our classification model. Overall, EfficientNet-B1 is the most desired base model for VectorBrain since it can easily run on basic smartphones due to its efficiency with a smaller number of parameters and model size while still achieving competitive performance.

### Visualization and interpretation of CNNs

Convolutional Neural Networks have proven to be very effective in image classification tasks, often exceeding human performance. However, as the complexity of these algorithms increases, especially in the case of deep networks, the models start to have hundreds of layers and thousands of training parameters. Deep Neural Networks appear to be ”black boxes,” taking in inputs and producing results quite accurately without providing any insight into their inner workings. This makes analyzing, debugging, and developing confidence in the model performance challenging. Bias could be included in the model performance due to changes in background texture/lighting conditions of the mosquito images used for training. Even variations in the dust on camera lenses can come up to become a source of bias in identifying different species. Due to this, verifying that the high accuracy that is being achieved on mosquito classification is being done based on morphological features of the specimen - and not other areas of the image—is of utmost importance.

#### Gradient weighted class activation map (Grad-CAM)

**Fig. 5:**
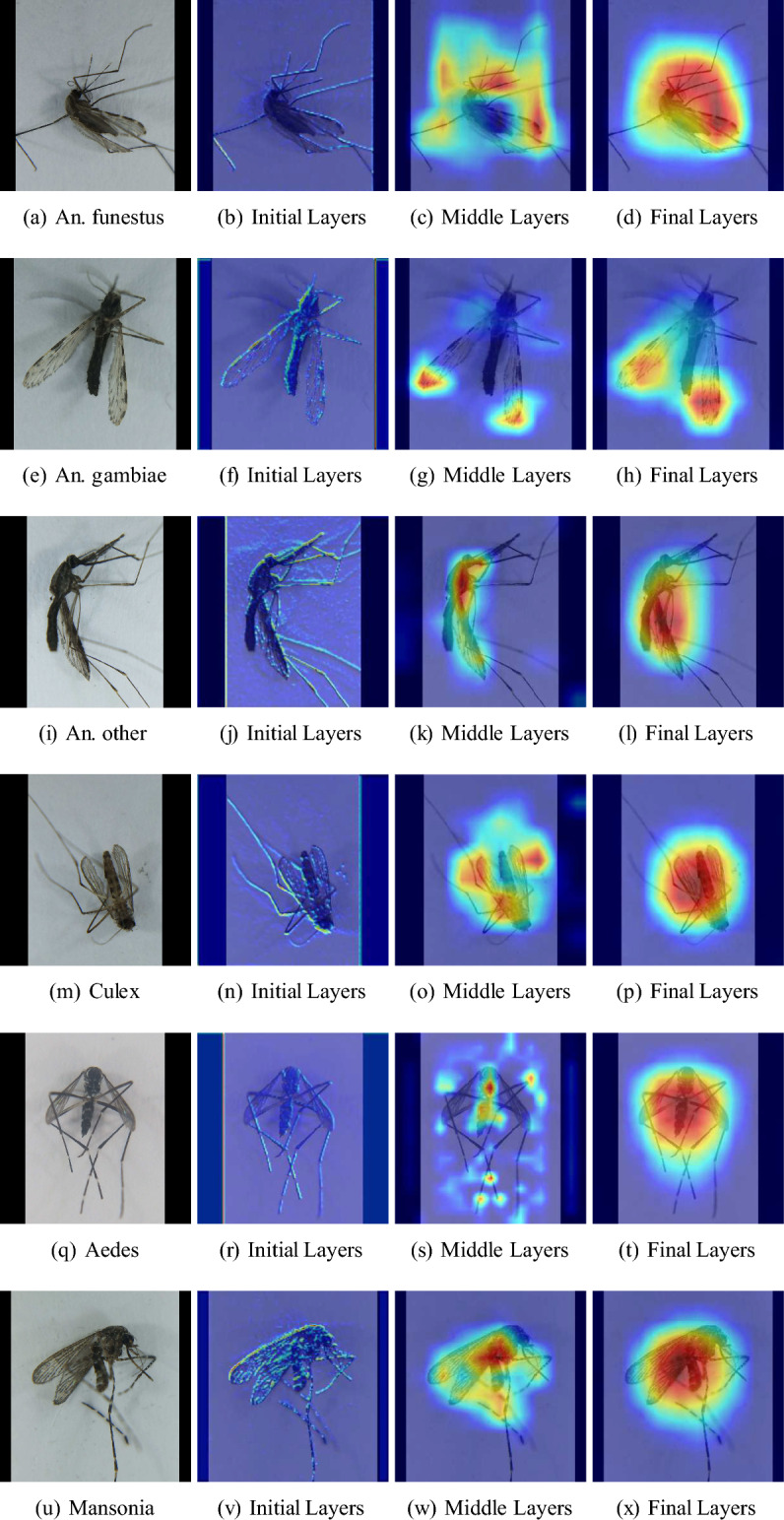
GradCam visualizations across layers to see what the model is learning. It is observed that the focus of the model is on the mosquito, ignoring the background whitespace. Specifically, regions of interest are observed to be the wings, legs, and body of the mosquito, which indicates that the model mimics the actions of a human expert.

To verify that the model is evaluating the mosquito’s morphology, Grad-Cam visualizations are used. Gradient weighted Class Activation Map (Grad-CAM)^31^ produces a heat map that highlights the important regions of an image by using the gradients of the target (mosquito species) of the final convolutional layer. Because the features go from low-level to high-level as the layers stack, visualizations of layers at different places of the network can provide more insights into how the network “perceives” the features at different levels using GradCam. From Fig. 5,it is clear that the network is looking at the different parts of the mosquito for distinct species. Specifically, each row presents an example of one of the 5-class species from the test data. Based on the second column in the figure, which is the visualization of the initial layers, the network extracts fine-grained features exclusively from the mosquitoes, spanning their entire body. These fine-grained features are aggregated to show a more informed interest area when going to the middle and final layers. In the first row, Figure (c) indicates that the network focuses on the wing pattern, especially towards the end, and legs the *An. funestus* specimen. Moreover, in the second row, the network focuses specifically on the tip of the wing pattern of the specimen, which is the crucial feature difference between *An. funestus* and the *An. gambiae*^6,7^. For the *An. other*, *Culex*, and *Mansonia* specimens, the network emphasizes the body, thorax, leg, and wings to make a joint decision. Analysis of visualizations of CNNs provides an interpretation of how the network works and understands the image, therefore, adding credibility to our approach.

#### Uniform manifold approximation and projection (UMAP)

**Fig. 6:**
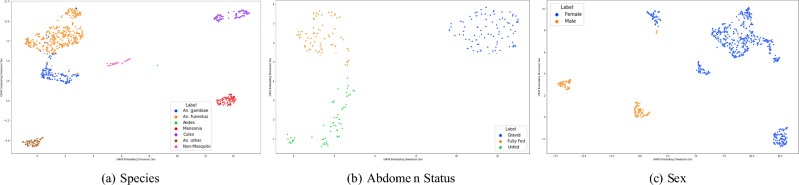
UMAP visualizations of the feature spaces for species, abdomen, sex classifications. The features are extracted from the layer before the last output layer.

Uniform Manifold Approximation and Projection (UMAP) is a dimensionality reduction technique, designed to uncover the underlying structure and relationships within high-dimensional data as visualizations. UMAP differs from traditional methods like Principal Component Analysis (PCA) and t-distributed Stochastic Neighbor Embedding (t-SNE) by preserving both local and global data structures, which helps maintain the integrity of data clusters and neighborhood relationships. This makes UMAP particularly valuable for tasks such as clustering, visualization, and pattern recognition, as it can reveal non-linear patterns and capture intricate data relationships effectively. For each of the species, sex and abdomen models , we extract the last feature map before the classifier head to get high dimensional features. From Fig. 6, we can see that for the test data, for each of the classification task, the points belonging to the same class appear to be clustered together, which reflects the network has learned similar representations for images belonging to a particular class. In the species plot, each class is separated into clusters, with the An. funestus and An.gambiae clusters being close to each other, highlighting the morphological similarities inherent in these species, making it harder for the model to differentiate between them. Also, we see for the sex plot that even though classes has multiple small clusters, they are differentiated by two sides; we can attribute this to the fact that the image features differ for each mosquito species as well as due to the global structure preserving property of UMAP. Gravid mosquitoes are placed farer in distance from unfed and fully fed mosquitoes in the abdomen status plot, which is likely because of the distinct white appearance.

### Evalutation of training and inference strategies

Training a neural network with a large amount of data can be time-consuming and computationally expensive. Therefore, we used transfer learning in our approach. Our previous work created a mosquito classification model by fine-tuning an ImageNet pre-trained model. We replicated this approach again with the EfficientNet architecture, and the resulting model was named MosquitoNet. The MosquitoNet model developed in this project is open-source. By making the model publicly available, other researchers can build upon and improve the model, leading to advancements in the field of mosquito classification and ultimately contributing to the fight against mosquito-borne diseases. The MosquitoNet model served as an ideal starting point for fine-tuning images captured on mobile phones. To fine-tune the MosquitoNet model for species, sex, and abdomen status classification, we trained the model on mobile phone images. We used various image augmentation techniques, such as horizontal flip, vertical flip, and rotations, to ensure the model’s robustness. Changes were made to the image’s saturation and exposure, but no random noise was added. The process of fine-tuning MosquitoNet for classification is described in more detail in the Method section. From the last two rows of Table 4, using transfer learning from MosquitoNet increases the accuracy by 2% while reducing the training time by 27% (1.2 h) for each fold on average.

**Table 4:** Accuracy (micro-averaged F1 score), macro-averaged F1 score, and weighted-averaged F1 score of mosquito classification under different training strategies in species on 5-fold cross-validation.

Strategies	Accuracy (avg (min–max))	Macro-averaged F1 score (avg (min–max))	Weighted-averaged F1 score (avg (min–max))
Species w/o detection and transfer learning	87.34 (85.90–88.50)%	86.70 (84.64–87.25)%	87.00 (86.07–88.10)%
Species with transfer learning only	89.40 (88.10–90.80)%	89.10 (87.43–90.20)%	89.52 (87.12–90.10)%
Species with detection only	92.40 (89.10–92.80)%	91.10 (90.43–93.20)%	92.40 (80.12–93.20)%
Species with all	94.44 (93.86–96.04)%	94.10 (92.43–95.20)%	94.40 (92.12–96.00)%

Even in a well-controlled setting, due to the small size of mosquitoes, a large portion of images will contribute to the white background and noise that will not be useful in classification tasks or could even distract the model. Cropping the image also maximizes the effective resolution of the mosquito since it eliminates the surrounding background and focuses solely on the mosquito, improving more than 5% in accuracy. This approach enables our model to capture detailed information on the mosquito’s morphology, including wing and leg features—essential for accurate mosquito species classification based on the well-developed dichotomous keys, Therefore, our approach leverages the advantages of mobile imaging while also addressing the challenges it presents.

**Fig. 7:**
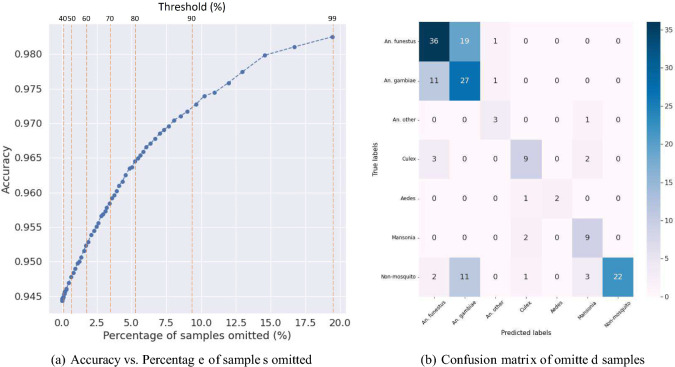
Plot (**a**) shows Accuracy vs. Percentage of samples omitted as a function of cutoff threshold in 1% increments from 0% to 99%. Plot (**b**) shows the confusion of the matrix of omitted samples in the count.

After the softmax layer, the scores from the output of the network are converted to normalized probabilities across classes, adding up to one for each sample. The index of the maximum probability corresponds to the predicted label. The confidence cutoff threshold, applied to this maximum probability, is introduced to remove the less confident candidate samples. The test sample is considered if the confidence value is above the threshold. Otherwise, it is omitted. This approach allows us to filter out the samples that are hard for the classifier, which might be due to image quality or extreme occlusions of mosquito body parts that can lead to the correct prediction. Based on Fig. 7, there is a tradeoff between accuracy and the percentage of omitted samples. The accuracy increases from 94.44 to 98.42% as more samples are omitted. We also find the results of more omitted samples being Anopheles, which reinforces our hypothesis that the model struggles more with the fine-grained classifications within the Anopheles genus. We manually checked the omitted samples and observed that 30% were blurry in different grades, and many were missing body parts (legs or wings, likely due to the incidents during the catching and imaging process). Therefore, the thresholding concept is effective in filtering out the candidates in the lower-quality images and leads to more trustworthy results in the field. The principle we are using to find the appropriate threshold is to control the percentage of omitted samples under 10%. During the imaging process, if a sample is determined as not confident, the user can flip the mosquito and retake an image. It is also worth noting that this strategy could be useful in novelty detection. If the data distribution of a novelty class is different from trained classes, the model is likely to “struggle” with the decision, causing a low confidence output.

## Discussions

To improve vector surveillance by automating mosquito identification, this work is the first to design and develop a deep learning pipeline based on lightweight CNNs with high accuracy that can be directly integrated with the current malaria vector surveillance programs in African countries. We aim to tackle the current obstacles in the available literature on machine learning and computer vision methods for mosquito identification. The strengths of this work include: (1) VectorBrain has shown a $94.44\pm 2\%$ species classification accuracy on the large-scale mosquito data set collected from the field, which is composed of freshly caught specimens imaging under basic smartphones during the daily vector surveillance activities. Therefore, we bridge the gaps between colony-bred specimens and wild-caught specimens and between high-resolution microscope/DSLR camera images and images taken with phones paired with an attached macro lens and portable imaging device. (2) Not only does VectorBrain have a competitive performance for mosquito species identification, but also for mosquito sex and abdomen status classification, with an accuracy of $97.66\pm 1\%$ and $82.20\pm 3\%$, respectively, replicating the complete results submitted to MoH for making vector control strategies. (3) VectorBrain is designed and centered with lightweight structures, opening the possibility of deploying on low-cost devices with memory constraints and limited computing power. Furthermore, running on the Android app provides a much more scalable solution and allows the users to obtain almost instantaneous results and feedback from the analysis of mosquito images.

During data collection, it is a common practice that the same specimen is imaged multiple times to obtain more sample points. However, similar photographs taken of the same specimen can be mixed between training and testing if following a simple image-wise splitting. Because the specimens could be uniquely damaged during the trapping process (such as wing scale or limb loss), as well as with the specific head/thorax/abdomen angle or fixed pose of dried specimens, the network may memorize and overfit to these information for prediction. Most of previous works are likely to follow this image-wise splitting based on their described strategy without further details. This overfitting problem is not obvious when verifying an offline algorithm, but will have implications on accuracy when testing on field-caught specimens, especially in a prospectively deployed field tool or application. Therefore, we propose a specimen-wise splitting that all images of a single specimen go to either the training or testing set, so there is no mix-up between them. In this way, the network is less prone to focus on irrelevant details and more generalizable to unseen specimen. The results of the algorithm reported here can be translated into a field-deployable tool for use by vector surveillance programs in Africa, which is the focus of ongoing work.

Deep learning methods usually perform well for in-distribution of data that they are trained on. Even though our algorithm has been trained on a large set of freshly caught mosquito images and proven to be effective across different districts and countries, out-of-distribution data could still pose challenges to algorithm accuracy. As a result, algorithm degradation is plausible when moving to different geographical locations or during different seasons in a year given variations within a mosquito species. To address this issue, timely finetuning can enable the deep learning models to continuously maintain above 90% accuracy with newly collected mosquito images. Going forward, we hope to explore the robustness of unseen data distributions and create a standard of practice to calibrate the model to new domains with as minimal samples as possible. While finetuning is an effective measure, the retraining process could be time and resource-consuming if simply adding new data to the complete dataset. On the other hand, finetuning with only newly collected data may cause a bias in the model that leads to “forgetting” what was learned before. Therefore, exploratory work in continuous learning, which aims to update the model’s knowledge with each labeled data point, can lead to an autonomous process to maintain algorithm accuracy.

The VectorBrain algorithm, also, is trained on specimens collected from a particular region of East Africa. As with all black box algorithms. It is not possible to determine if the features of the mosquito used to differentiate between these species are the same as the ones used by human experts universally. As a result, it is possible that this model might have overfitted to some morphological features that are unique to the mosquitoes in East Africa, although this is unlikely. Future work should validate the ability of this algorithm to generalize specimens of these species collected from across multiple geographies. In this study, we only looked at three categories of Anopheles mosquitoes which are the main vectors in East Africa. However. If this algorithm is used in multiple geographies, it would need to be trained for many other anomaly species prevalent to those geographies. Depending on the differences between morphological features between these Anopheles species, the algorithm accuracy might be different from what we were able to accomplish here with a limited subset of species. However, our past work^20^ has shown that up to 39 species can be differentiated with accuracies as high as 97%.

The currently proposed pipeline has the potential to enable automated community-based vector surveillance in rural African regions, which presents several significant advantages in transforming malaria disease control efforts. This approach provides a cost-effective means of achieving enhanced spatial and temporal resolution of vectors in the environment, particularly in areas with limited bandwidth. Furthermore, this paradigm shift in surveillance capitalizes on the principle of task-sharing, delegating responsibilities to health workers who are intrinsically motivated by the well-being of their communities. The abundance of health workers truly is an untapped potential of the surveillance workforce, especially when compared to the limited number of field entomologists in countries like Uganda. By facilitating community-based vector surveillance through digital tools, health workers can collect and report data more efficiently, fostering rapid validation and adaptation of vector control strategies. Thus far, the performance of the model was achieved in a controlled efficacy study with experiments ran in the lab. However, in parallel to our efforts creating the VectorBrain architecture, we conducted and analyzed findings from a summative usability study with village health teams from rural towns in Uganda as study participants^32^. This usability study presents a combined hardware and software solution called VectorCam, which incorporates the VectorBrain architecture locally onto a smartphone in low-resource settings. VectorCam was designed with human-centric design principles to optimize for a VHT workload and in low bandwidth areas. Overall, the study benchmarked VectorCam as a usable device by the VHTs via standardized system usability scale questionnaires and assessing the timeliness of surveillance using this new paradigm. Ultimately, we hope to validate this digital platform through an ongoing longitudinal field effectiveness study to assess AI performance and conductance of vector surveillance through non-expert community members.

Our algorithms significantly enhance the capabilities of field entomologists in mosquito identification, by empowering them to become vector control strategists by opening more time for data analysis and response. This advancement enables more extensive vector surveillance and increases the throughput of mosquito identification in the hands of a non-expert without sacrificing accuracy. Crucially, our approach offers instantaneous identification in the field, mitigating the risk of misclassification due to morphological changes when specimens are transferred from collection sites to distant laboratories. By ensuring accurate and timely identification with trackable data management, our method supports the need for data-driven interventions and facilitates more effective monitoring and evaluation of mosquito populations. The implementation of AI enabled digital tools such as VectorBrain is a promising approach to disrupt the current paradigm of malaria control in remote regions of the world, giving opportunities to advance preventative measures in these communities.

## Methods

### Datasets

**Table 5:** Dataset Description—Unless specified, the counts of the number of specimens are reported. The classes in the Species column are *An. funestus*, *An. gambiae*, *An. other*, *Culex*, *Aedes*, *Mansonia*, and *Non-mosquitoes*. The classes in the column of Sex are Female and Male (F/M). The classes in the column of abdominal status are unfed, fully fed, and gravid (UF/FF/G). The gold standard for species was based on PCR or molecular sequencing. Sex and Abdominal Status was based on morphological identification done by local vector control officers (VCOs) using a light microscope.

Dataset	Specimens/images	Imaging modality	Species	Sex	Abdominal status	Origin
MosquitoNet	2696/24052	Miscope, DSLR camera	42 species	N/A	N/A	Multiple African and Asian countries^20^
VectorCAM	4195/11532	Phone+macro lens	1570/968/427/748/12/286/184	2477/438	1093/426/329	Uganda, Ghana, Zambia

Our computer vision algorithm—VectorBrain, was sequentially trained using two different datasets obtained over different time periods and geographies. We refer to these datasets as MosquitoNet dataset and VectorCAM dataset as summarized in Table 5. Imaging devices include MiScopes (Zarbeco Inc.), DSLR cameras (Nikon, Canon, various models), and Android smartphones (various manufacturers/models) with clipped-on 15X macro lenses. These datasets are collected in different countries, districts, and collection sites to account for possible variations in morphology within species, such as size, hue, shapes, etc. due to environmental differences and aging^33^. Incorporating all these datasets into the training process will lead to more robust model and achieve better generalization based on geographical locations. The descriptions of the datasets are given below:

- MosquitoNet Dataset: A diverse image database that is primarily composed of desiccated mosquito specimens from field sites across Africa and North America, with the supplement of lab colony specimens from species that are difficult to obtain in the field. To identify each mosquito, the genetic identification that was either DNA-barcoding comparison against the gold-standard Mosquito Barcoding Initiative reference library performed by Walter Reed Biosystematics Unit (WRBU) or using specific nuclear DNA ITS2 assays was used when available. Otherwise, the mosquito would have been identified by human experts under a microscope. Specimens were photographed using the USB microscope (Zarbeco MiScope Handheld Digital Micro-scope-MiSC-s2) set at 40X zoom on a white background with the resolution being 4 microns, for some of which DLSR cameras were also used to take images. Each mosquito was flipped multiple times to produce 4–10 images per specimen. In total, the database has 24,052 photos of 2696 specimens from 67 species. Further details about the data set and breakdown of the number of images for each species are included in our previous paper^20^. This dataset was used to pretrain the VectorBrain CNN before transfer learning using the VectorCam dataset.
- VectorCAM Dataset: This data set consists of images taken using Android phones with a macro lens in the field during routine vector surveillance. The smartphones are clipped-on with a macro lens to achieve an optical resolution of 57 Lp/mm (Line Pairs per Millimeter) for imaging and a depth of field that keeps the majority of the mosquito body in focus. This was paired with custom-designed hardware that maintained a uniform lighting environment using LED strips, and a uniform imaging distance from the lens to the mosquito. Each specimen has been imaged 3–5 times from different angles to view the wing patterns, abdomen status, markings on the legs, etc from all directions. These images were imaged in districts of Uganda (including Adjumani, Soroti, Lira, Busia, Gulu, Mayuge, and Bugiri), Ghana (including Tamale and Walewale, and Zambia (including Macha and Choma). All images were taken as per approved IRB protocol from the Johns Hopkins University Institutional Review Board (IRB), as well as respective IRBs in Uganda (TASO, and Makerere University School of Public Health), Zambia (Macha Research Trust), and Ghana (University of Ghana—Noguchi Memorial Institute for Medical Research).

### Model architecture

#### YOLO v5 small

YOLO v5^25^ is a single-stage detector that consists of three parts: 1. a backbone—Cross Stage Partial Network (CSPNet)^34^, which improves gradient flow and accuracy with fused features depending on anchor size, to extracts features from the input image 2. a neck Path Aggregation Network (PANet)^35^ that aggregates features from different scales 3. a head to predict object bounding boxes, class probabilities, and objectness scores using the features from the neck. Moreover, YOLOv5 uses data augmentations, such as scaling, color space adjustments, mosaic augmentation, and self-adversarial training, which involves training the model on a large dataset of labeled images and then using the model to generate new pseudo-labeled data. The model is then retrained on this new data, and the process is repeated iteratively to improve performance. Overall, YOLO v5 is an effective and efficient object detection algorithm that handles the detection phase of our proposed pipeline. The comparative analysis with other object detection models on the number of parameters, size, speed, and accuracy is listed in Table 2.

**Fig. 8:**
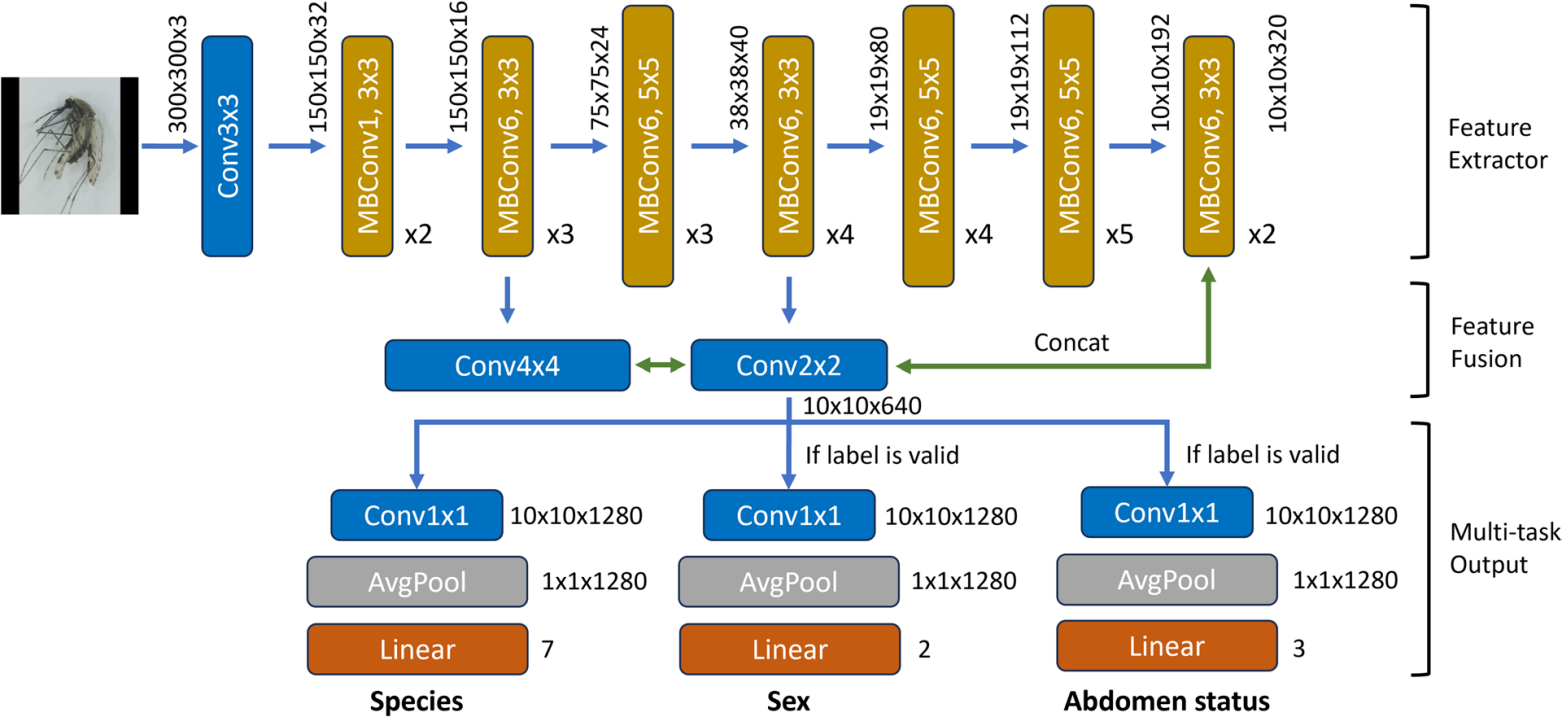
Our uniquely designed multi-task network with an EfficientNet backbone.

#### VectorBrain

VectorBrain uses EfficientNet as its backbone, which was introduced by Tan et al^26^ to achieve better accuracy using fewer parameters and less computation. A novel compound scaling method uniformly scales all network dimensions (depth, width, and resolution) with fixed scaling coefficients. This allows the network to be more flexible and balance the trade-off between model complexity and computational efficiency. At the end, VectorBrain needs to run locally on smartphones, so it is crucial to consider the model’s accuracy and portability. EfficientNetb1 is particularly advantageous due to its lightweight architecture (7.8M #Params and 0.70B #FLOPS), yet with high accuracy. Our uniquely designed network architecture, inspired by Hyperface^36^, is illustrated in Fig. 8. The upper part of the architecture is the feature extractor, in which we keep the layers of pretrained EfficientNet, the MosquitoNet we called, up to the last layer before the output header. Then, the features from the early, middle, and later layers are fused together to form a more comprehensive representation, incorporating low-level to high-level information. This is beneficial, especially in the classification between * An. funestus* and *An. gambiae*, as the differences are fine-grained details, which could be detected with low-level features. At the final stage, the network is split into three branches, each comprised of a convolutional block to increase its expressive power, an average pooling layer to downsample the feature map, and a fully connected layer to produce an output equal to the number of classes in each task. A unique challenge in training strategy design is that not all specimens have all three labels, such that abdominal status is trivial for male mosquitoes, and inclusion of a broad heterogeneous class such as *Non-mosquitoes* that does not need further sex or abdomen status classification. Therefore, we control the activation of branches in the final stage separately during training by assigning a special number to the invalid label. Whenever this special number is present, the features after fusion do not pass through the corresponding output branch so that its weights do not get updated, shown in Fig. 8 lower part. The features still pass through the branch where the label is valid. During inference, the image is analyzed through all three branches, and the interested ones are kept for analysis.

### Experimental details

ImageNet^37^ contains 14,197,122 annotated images spanning thousands of categories, which has served as an influential benchmark for image recognition algorithms. An ImageNet pre-trained model has the advantages of improved performance and faster convergence during transfer learning especially when data is limited. It works well mostly on natural images but has little contribution to our use case due to the large domain difference where the images of mosquitos are much less variant in color. MosquitoNet is our equivalent pre-trained “ImageNet” model, trained with 12,977 photos of 2696 specimens from 42 mosquito species, providing an excellent starting point to transfer learning to the phone imaging based database. Using a pre-trained model, we could leverage the knowledge and representations learned by the model to adapt better in the domain and improve the performance of our multi-task EfficientNet.

Maintaining specimen-level segregation throughout the training and testing phases is a critical aspect of our study, as it contributes to reliable and robust results. We employed a 5-fold stratified cross-validation to divide the dataset into folds of three non-overlapping sets: training set, validation set, and testing set. The percentage of samples in each class is preserved in each fold. Each specimen may have multiple images captured at different angles or under different lighting conditions. We ensure that images from the same mosquito are not present in different sets, as this can lead to overfitting and over-optimistic model performance. Without specimen-level segregation, there is a risk of overestimating model performance and misinterpreting the results. This is because mosquitoes from the same specimen can have similar morphological characteristics, and including them in both the training and testing sets can artificially inflate the model’s accuracy. Maintaining specimen-level segregation allows us to more accurately evaluate the performance of our models on unseen mosquitoes, as in real-world scenarios.

To address the overfitting issue and the vanishing gradient problem, we utilized the Weighted Adam (AdamW) optimizer^38^, incorporating weight decay into the Adam adaptive learning rate algorithm. We used a learning rate of 0.005, which was found to be the optimal value through a grid search of several learning rates. The model was trained for 100 epochs with early stopping used to prevent overfitting, which triggered if there was no improvement in validation accuracy for 10 epochs, and the model with the best validation accuracy was saved. During training to account for different image conditions that might be encountered during the deployment, the images were randomly augmented to improve the robustness of the model to changes in lighting, orientation, and other factors. Specifically, each image was randomly flipped horizontally and vertically, rotated by up to $90^\circ$, and zoomed in or out by up to 10%. Additionally, random brightness and contrast adjustments were applied to each image. The loss function used during training was categorical cross-entropy, commonly used for multi-class classification problems^39^. During each epoch, the loss between the true labels and the predicted probabilities was minimized through backpropagation, which updates the model weights to reduce the loss. Overall, the models were trained using a carefully chosen combination of hyperparameters, data augmentation techniques, and optimization algorithms to achieve state-of-the-art performance on mosquito identification tasks.

Loss for a multi-class classification^40^ problem is defined as:

1 $$\begin{aligned} \mathscr {L}_{\text{ log } } = -\frac{1}{N}\sum _{i=1}^{N}\sum _{j=1}^{C}y_{ij}\log (\hat{y}_{ij}) \end{aligned}$$

where $\mathscr {L}_{\text{ log } }$ is the log loss, *N* is the number of samples, *C* is the number of classes, $y_{ij}$ is the true label of the *i*-th sample for the *j*-th class (which is 1 if the sample belongs to that class and 0 otherwise), $\hat{y}_{ij}$ is the predicted probability of the *i*-th sample for the *j*-th class, and $\theta$ represents the model parameters.

Our goal was to train our multi-task EfficientNet, illustrated in Fig. 8, for species, sex, and abdomen classifications at the same time. The log loss is computed individually for each task, and the total objective loss is:

2 $$\begin{aligned} \mathscr {L}_{\text{ total } }=\lambda _{1} \mathscr {L}_{\text {species}}+\lambda _{2} \mathscr {L}_{\text {sex}}+\lambda _{3} \mathscr {L}_{\text {abdomen}} \end{aligned}$$

where $\lambda$s are corresponding weights.

PyTorch was used to build, train, and validate the models throughout the processes. PyTorch data loaders and transforms were employed to enable parallel data loading and preprocessing for efficient data handling. Additionally, a weighted sampling algorithm was implemented to alleviate the class imbalance problem during training. Let *N* classes and $n_c$ be the number of samples in class *c*, where *c* ranges from 1 to *N*. The weight of each class is defined as the inverse of the number of samples in the class:

3 $$\begin{aligned} w_c = \frac{1}{n_c}, \hspace{0.5cm} w_c = \frac{w_c}{\sum _{i=1}^N w_i}, \hspace{0.5cm} p_c = \frac{w_c}{\sum _{i=1}^N w_i} \end{aligned}$$

We can then normalize the weights so that they sum to one, where $\sum _{i=1}^N w_i$ is the sum of all the weights. The probability of selecting a sample from class *c* is proportional to the weight of the class $w_c$. In practice, the sampler selects samples based on their corresponding class probabilities $p_c$ computed above, resulting in an approximately balanced distribution of different classes in each training batch. All training and validation for MosquitoNet and its fine-tuned versions were conducted in-house on two NVIDIA RTX 2080 Ti GPUs, each with 11GB of VRAM.

### Confidence cutoff levels

To enhance the real-time performance of the model for a better user experience, we introduced a thresholding concept. This is achieved by comparing the confidence values generated by the model for a specific test sample with a fixed float threshold value. The output of the model is set by varying the values passed through a softmax function, which produces probability values summing to 1, and each value is between 0 (least confident) and 1 (most confident). To obtain the confidence value, the maximum of the output probabilities is selected. If the confidence value is above the threshold, the test sample is considered. Otherwise, it is ignored and deemed not confident. The threshold value is set to 0.8 empirically, taking into consideration the balance between not ignoring too many specimens and not incorrectly classifying too many. The general criterion is that the number of dropped samples takes no more than 10% of the entire test set. Using the thresholding strategy, the test samples that confuse the model or are of low quality (blurring or mosquito parts occlusions due to angles or damage during collection) could be ignored.

4 $$\begin{aligned} y = {\left\{ \begin{array}{ll} 1, & \quad \text {if } \times \geqslant T \\ 0, & \quad \text {otherwise} \end{array}\right. } \end{aligned}$$

In this equation, *x* represents the confidence value of a given test sample, and *T* represents the threshold value. If *x* is greater than or equal to *T*, the output *y* is 1, indicating that the sample is considered as confident. Otherwise, the output *y* is 0, indicating that the sample is not considered as confident.

### Evaluation metrics

Accuracy, precision (or sensitivity), recall, and F1 scores^41^ are used throughout this paper to evaluate the performance of our models. The F1 score is the harmonic mean of the precision and recall and given by the following equations:

5 $$\begin{aligned} \text {F}_{1} = 2 \times \frac{\text {precision} \times \text {recall}}{\text {precision} + \text {recall}} \end{aligned}$$

where the precision and recall are defined as:

6 $$\begin{aligned} \text {precision} = \frac{\text {true positives}}{\text {true positives} + \text {false positives}}, \hspace{0.5cm} \text {recall} = \frac{\text {true positives}}{\text {true positives} + \text {false negatives}} \end{aligned}$$

The weighted-average F1 score scales the individual class’s F1 score taking into account the samples in each class. To account for the imbalanced class distribution, where *Anopheles gambiae* has fewer samples but high consideration, macro-averaged F1 score is also used to evaluate the results. It is calculated by computing the F1 score for each class and then taking the average of the scores. The formula for weighted-average F1 and macro-averaged F1 score are:

7 $$\begin{aligned} F_{1weighted} = \sum _{i=1}^{C} \frac{N_i \cdot F_{1i}}{N}, \hspace{0.5cm} F_{1macro} = \frac{\sum _{i=1}^{C} F_{1i}}{C} \end{aligned}$$

where *C* is the total number of classes, N is the total number of samples, and the subscript *i* represents the *i*-th class.

## Data Availability

Data collected and analyzed in this study is available from the corresponding author upon reasonable request.

## Code availability

The open-source codebase is available in the GitHub repository: https://github.com/VecCam/VectorBrain.
